# The novel proteasome inhibitor carfilzomib activates and enhances extrinsic apoptosis involving stabilization of death receptor 5

**DOI:** 10.18632/oncotarget.3947

**Published:** 2015-05-11

**Authors:** Bo Han, Weilong Yao, You-Take Oh, Jing-Shan Tong, Shaohua Li, Jiusheng Deng, Ping Yue, Fadlo R. Khuri, Shi-Yong Sun

**Affiliations:** ^1^ Department of Hematology and Oncology, Emory University School of Medicine and Winship Cancer Institute, Atlanta, GA, USA; ^2^ State Key Laboratory of Oral Disease and Department of Head and Neck Oncology, West China Hospital of Stomatology, Sichuan University, Chengdu, PR China; ^3^ Department of Pharmacology and Chemical Biology, University of Pittsburgh Cancer Institute and School of Medicine, Pittsburgh, PA, USA; ^4^ Beijing Institute of Basic Medical Sciences, Beijing, PR China

**Keywords:** proteasome inhibitors, carfilzomib, death receptor 5, extrinsic apoptosis

## Abstract

Carfilzomib (CFZ) is a second generation proteasome inhibitor approved for the treatment of patients with multiple myeloma. It induces apoptosis in human cancer cells; but the underlying mechanisms remain undefined. In the present study, we show that CFZ decreases the survival of several human cancer cell lines and induces apoptosis. Induction of apoptosis by CFZ occurs, at least in part, due to activation of the extrinsic apoptotic pathway, since FADD deficiency protected cancer cells from undergoing apoptosis. CFZ increased total and cell surface levels of DR5 in different cancer cell lines; accordingly it enhanced TRAIL-induced apoptosis. DR5 deficiency protected cancer cells from induction of apoptosis by CFZ either alone or in combination with TRAIL. These data together convincingly demonstrate that DR5 upregulation is a critical mechanism accounting for CFZ-induced apoptosis and enhancement of TRAIL-induced apoptosis. CFZ inhibited the degradation of DR5, suggesting that DR5 stabilization contributes to CFZ-induced DR5 upregulation. In summary, the present study highlights the important role of DR5 upregulation in CFZ-induced apoptosis and enhancement of TRAIL-induced apoptosis in human cancer cells.

## INTRODUCTION

Ubiquitin/proteasome-mediated protein degradation represents a critical post-translational mechanism for the regulation of protein levels. This process primarily involves polyubiquitination of substrate proteins and subsequent proteolytic degradation by the macromolecular 26S proteasome complex. It has been assumed that inhibition of the proteasome causes accumulation of certain proteins deleterious to the survival of cancer cells, allowing restoration of cell cycle arrest and/or apoptotic cell death [1, 2]. Thus targeting the proteasome has emerged as a promising cancer therapeutic strategy. The successful development of bortezomib, a first-generation proteasome inhibitor, as an FDA-approved anticancer drug has spurred efforts to identify and develop second-generation proteasome inhibitors with improved selectivity and therapeutic efficacy [2, 3].

**Figure 1:**
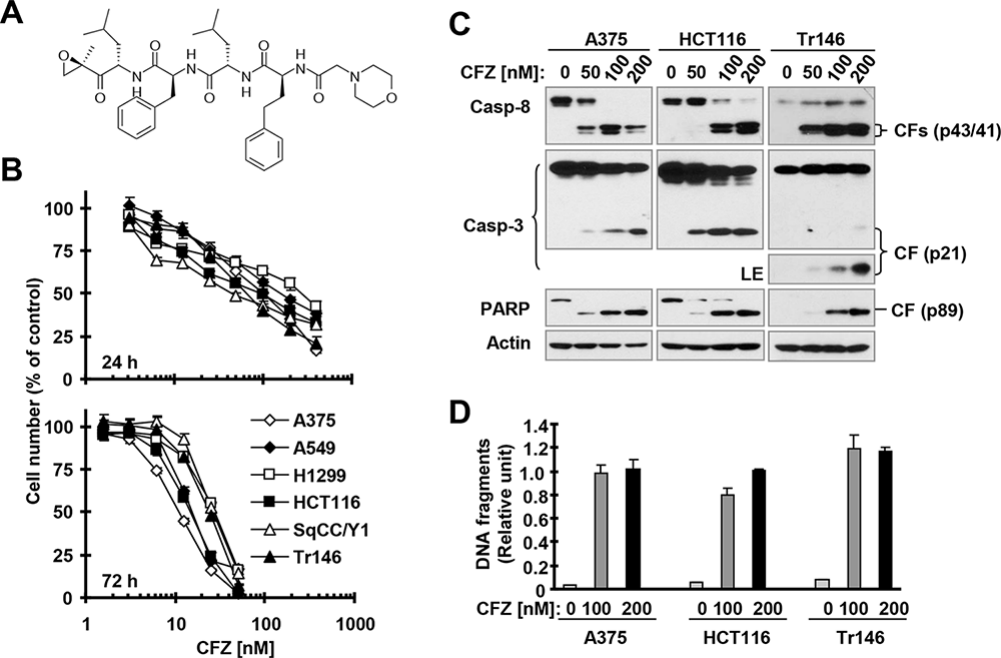
CFZ (A) effectively decreases the survival of cancer cells (B) and induces apoptosis (C and D) A, Chemical structure of CFZ. B, The indicated cancer cell lines were treated with different concentrations of CFZ for 24 or 72 h and then subjected to estimation of cell number with the SRB assay. The data are means ± SDs of four replicate determinations. C and D, The indicated cell lines were treated with the given concentrations of CFZ for 24 h and then subjected to preparation of whole-cell protein lysates and subsequent Western blot analysis (C) or ELISA assay to detect DNA fragments (D). The data are means ± SDs of triplicate determinations. CF, cleaved fragment; LE, long exposure.

Carfilzomib (CFZ; also known as PR-171), a second-generation irreversible proteasome inhibitor, is approved by the FDA for the treatment of multiple myeloma. It is a cell-permeable tetrapeptide epoxyketone analog of epoxomicin (Figure 1A) that is structurally distinct from bortezomib and irreversibly binds to and inhibits the chymotrypsin-like site of the proteasome [4, 5]. In addition to hematologic malignancies, CFZ is also being evaluated in clinical trials against solid tumors including small-cell lung cancer, non-small cell lung cancer, refractory renal cell cancer, and metastatic prostate cancer [2].

Preclinical studies have shown that CFZ induces apoptosis in cell lines derived from multiple myeloma [6], Waldenstrom's macroglobulinemia [7], chronic lymphocytic leukemia [8], lymphoma [9, 10], head and neck cancer [11] and lung cancer [12]. CFZ-induced apoptosis in different cancer cells has been suggested to be associated with c-Jun N-terminal kinase (JNK) activation [6, 7, 10] and Bik or Bak upregulation [9, 11]. Otherwise, the mechanisms by which CFZ induces apoptosis are largely unknown.

Apoptosis can occur through two pathways: the extrinsic apoptotic pathway that primarily involves signals transduced through death receptors and the intrinsic apoptotic pathway that largely relies on signals from the mitochondria. Both pathways involve the activation of caspase cascades, which in turn cause cleavage of cellular substrates and result in the characteristic morphological and biochemical changes constituting the process of apoptosis [13, 14]. The extrinsic pathway is characterized by the trimerization of cell surface death receptors and activation of caspase-8, while the intrinsic pathway involves the disruption of mitochondrial membranes, release of cytochrome c from the mitochondria, and the activation of caspase-9. One well-known death ligand is tumor necrosis factor-related apoptosis-inducing ligand (TRAIL), which initiates apoptosis upon ligation with two death receptors: death receptor 4 (DR4) and 5 (DR5). TRAIL preferentially induces apoptosis in transformed or malignant cells while sparing most normal cells and thus is a tumor-selective apoptosis-inducing cytokine with cancer therapeutic potential [15].

In this study, we determined the effects of CFZ on the induction of apoptosis in different solid tumor cell lines and were particularly interested in elucidating the mechanisms by which CFZ induces apoptosis in these cancer cells. We demonstrated that CFZ activates and enhances extrinsic apoptosis primarily through DR5 upregulation, in part due to delaying DR5 protein degradation.

## RESULTS

### CFZ effectively decreases the survival of different cancer cell lines and induces apoptosis

To determine the effective concentration range of CFZ that suppresses the growth of different cancer cell lines, we treated 6 cancer cell lines with varied concentrations of CFZ for 24 and 72 h, and then estimated cell numbers. We found that CFZ effectively decreased the survival of these cells with IC_50_s ranging from 50 nM to 300 nM (24 h exposure) and from 10 to 30 nM (72 h treatment) (Figure 1B). Clearly, prolonged treatment enhances the efficacy of CFZ in inhibiting cancer cell growth. We further determined the effects of CFZ on hallmarks of apoptosis in three cancer cell lines, specifically, cleavage (or activation) of caspases and PARP and induction of DNA fragmentation. We detected cleavage of caspase-8, caspase-3 and PARP as indicated by the appearance of cleaved forms of these proteins in A375, HCT116 and Tr146 cells exposed to CFZ for 24 h, demonstrating that CFZ activates these caspases (Figure 1C). In agreement, we detected increased levels of DNA fragments in cells treated with CFZ (Figure 1D), indicating that CFZ induces DNA fragmentation. Hence, CFZ clearly induces apoptosis in cancer cells, constituting an important mechanism accounting for its cell-killing effect.

### CFZ induces caspase-dependent apoptosis in part through activation of the extrinsic apoptotic pathway

**Figure 2:**
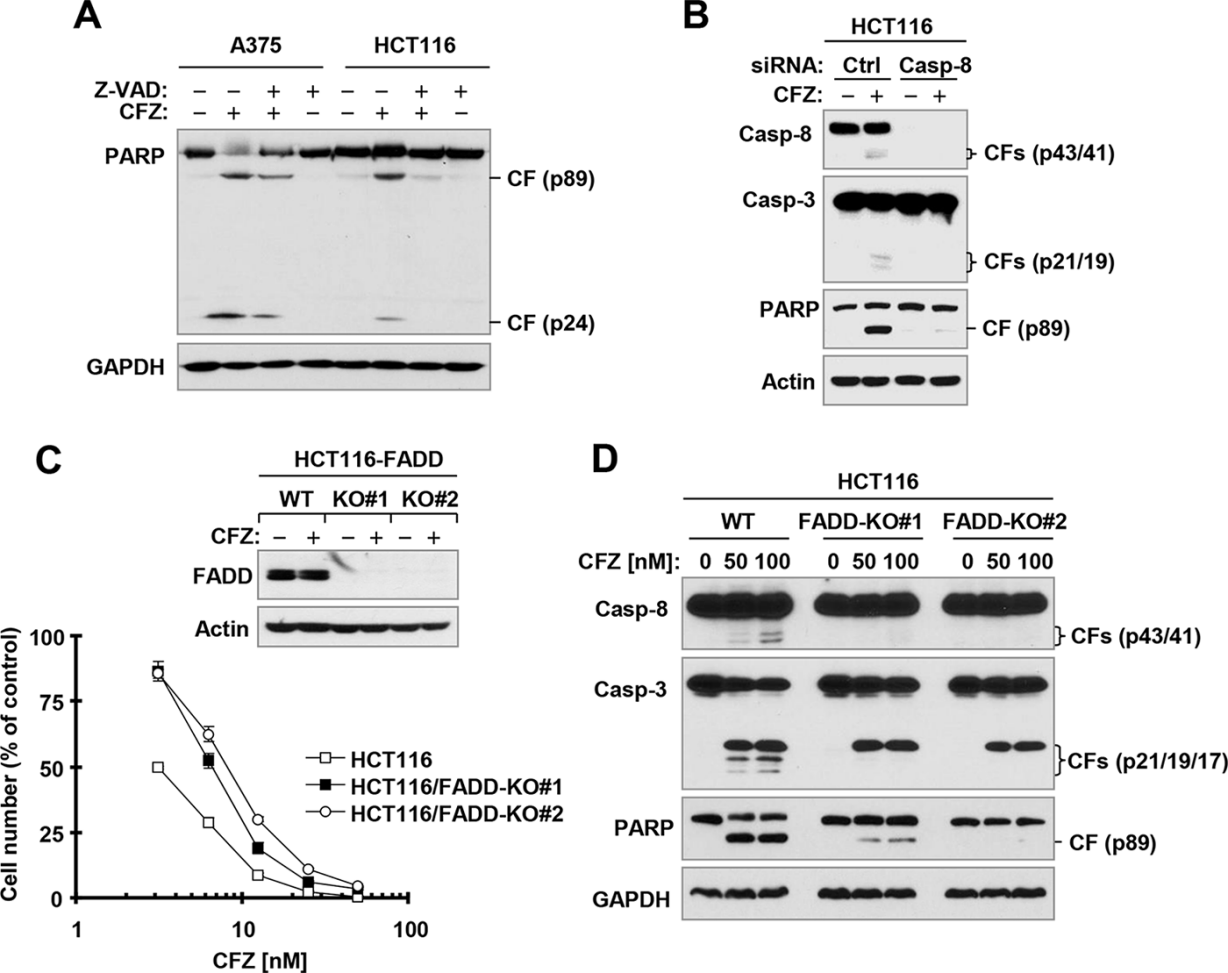
Caspase inhibition (A) caspase-8 knockdown (B) or FADD deficiency (C and D) protects cancer cells from CFZ-induced apoptosis A, The indicated cell lines were treated with 100 nM CFZ in the absence or presence of 20 μM Z-VAD-FMK for 24 h and then harvested for preparation of whole-cell protein lysates and subsequent Western blot analysis. B, HCT116 cells were transfected with the indicated siRNAs and then after 36 h, were exposed to 100 nM CFZ for an additional 24 h. The cells were harvested for preparation of whole-cell protein lysates and subsequent Western blot analysis. C, The indicated WT and FADD-KO cell lines were treated with 100 nM CFZ for 8 h and then harvested for preparation of whole-cell protein lysates and subsequent Western blot analysis to confirm FADD expression. The indicated cell lines were also treated with different concentrations of CFZ for 48 h and then subjected to estimation of cell number with the SRB assay. The data are means ± SDs of four replicate determinations. D, The given cell lines were treated with the indicated concentrations of CFZ for 17 h and then subjected to preparation of whole-cell protein lysates and subsequent Western blot analysis. CF, cleaved fragment.

The above data clearly show that CFZ strongly induces cleavage of, and thus activates, caspase-8, a well-known initiator caspase in the extrinsic apoptotic pathway. To examine whether activation of caspases, particularly caspase-8, is required for CFZ-induced apoptosis, we compared PARP cleavage in the absence and presence of the pan-caspase inhibitor, Z-VAD-FMK, and found that PARP cleavage by CFZ was substantially inhibited by Z-VAD-FMK (Figure 2A). When caspase-8 was silenced with caspase-8 small interfering RNA (siRNA), CFZ-induced cleavage of both caspase-3 and PARP were blocked (Figure 2B). These results indicate that CFZ induces caspase-8-dependent apoptosis. Moreover, we determined whether CFZ induces apoptosis through activation of the extrinsic apoptotic pathway. We compared the effects of CFZ on decreasing cell survival and inducing apoptosis in isogenic HCT116 cell lines with wild-type (WT) FADD and with FADD knocked out (FADD-KO). In the survival assay, both FADD-KO HCT116 cell lines were significantly more resistant than WT HCT116 cells to CFZ, although they were not completely resistant (Figure 2C). In agreement, CFZ induced strong cleavage of caspase-8, caspase-3 and PARP in WT HCT116 cells, but only weakly in FADD-KO cells (Figure 2D). Hence, it is clear that deficiency of FADD, an essential component in mediating extrinsic apoptotic signaling, partially protects cancer cells from induction of apoptosis by CFZ, indicating that activation of the extrinsic apoptotic pathway contributes to CFZ-induced apoptosis.

### CFZ upregulates the expression of TRAIL death receptors, particularly DR5, in cancer cells

**Figure 3:**
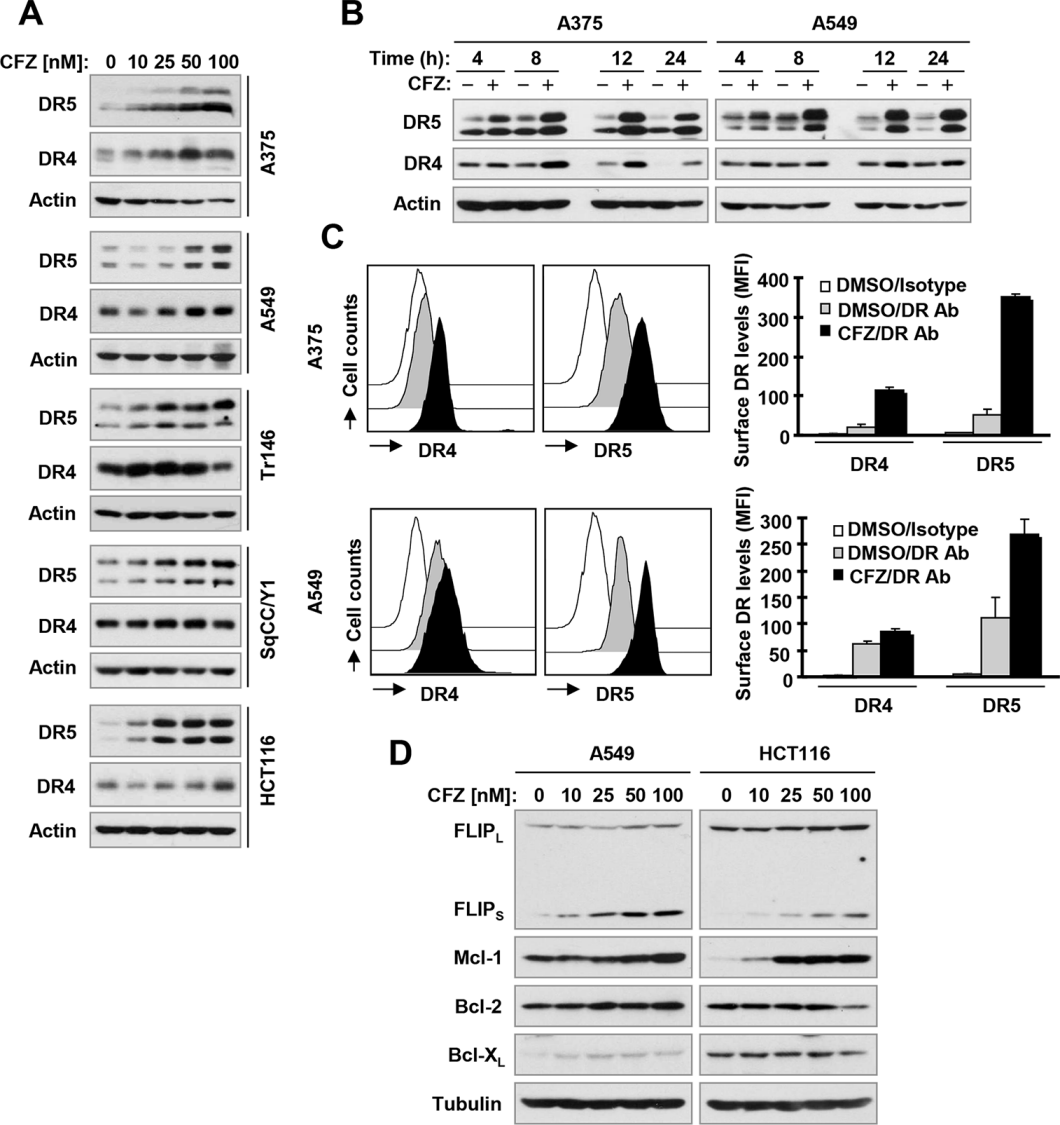
CFZ increases total (A and B) and cell surface (C) levels of DR5 and DR4 accompanied with elevated c-FLIP and Mcl-1 (D) A, B and D, The indicated cell lines were treated with the given concentrations of CFZ for 8 h (A and D) or with 100 nM CFZ for different times (B) and then subjected to preparation of whole-cell protein lysates. The given proteins were detected using Western blot analysis. C, The indicated cell lines were treated with 100 nM CFZ for 15 h and then harvested for analysis of cell surface DR5 and DR4 by immunofluorescence staining and subsequent flow cytometry. The open peak represents DMSO-treated cells stained with a matched control PE-conjugated IgG isotype antibody. The filled grey peaks show DMSO-treated cells stained with PE-conjugated anti-DR5 or DR4 antibody. The filled black peaks represent CFZ-treated cells stained with PE-conjugated anti-DR5 or DR4 antibody.

To understand the mechanism by which CFZ activates extrinsic apoptosis, we examined the effects of CFZ on the expression of DR4 and DR5, which are important cell surface death receptors that activate extrinsic apoptotic signaling though recruitment of FADD. At a concentration range of 25–100 nM, CFZ increased the levels of DR5 in a concentration-dependent manner in all of the tested cancer cell lines (Figure 3A). CFZ also increased DR4 levels somewhat in these cell lines, but the effects were either very weak (HCT116 and SqCC/Y1) or not concentration-dependent (Tr146 and SqCC/Y1) (Figure 3A). The increase in DR5 and DR4 expression in CFZ-treated cells occurred by 4 h, peaked at 8 h, and was maintained for up to 24 h (Figure 3B), indicating that CFZ-induced upregulation of DR5 and DR4 is an early and persistent event. Consistent with the greater increase in expression of DR5 compared with that of DR4 in CFZ-treated cells (Figure 3A), cell surface levels of DR5 were substantially increased by CFZ treatment while surface DR4 levels were only weakly increased (Figure 3C). Taken together, we conclude that CFZ strongly increases total and cell surface DR5 expression in cancer cells.

We further determined whether CFZ alters the levels of other proteins including c-FLIP, Mcl-1, Bcl-2 and Bcl-X_L_ that are known to inhibit apoptosis [14]. As presented in Figure 3D, CFZ clearly increased the levels of FLIP_S_ and Mcl-1 in a concentration-dependent manner in both A549 and HCT116 cells with limited effects on changing the levels of Bcl-2 and Bcl-X_L_.

### CFZ enhances TRAIL-induced apoptosis

**Figure 4:**
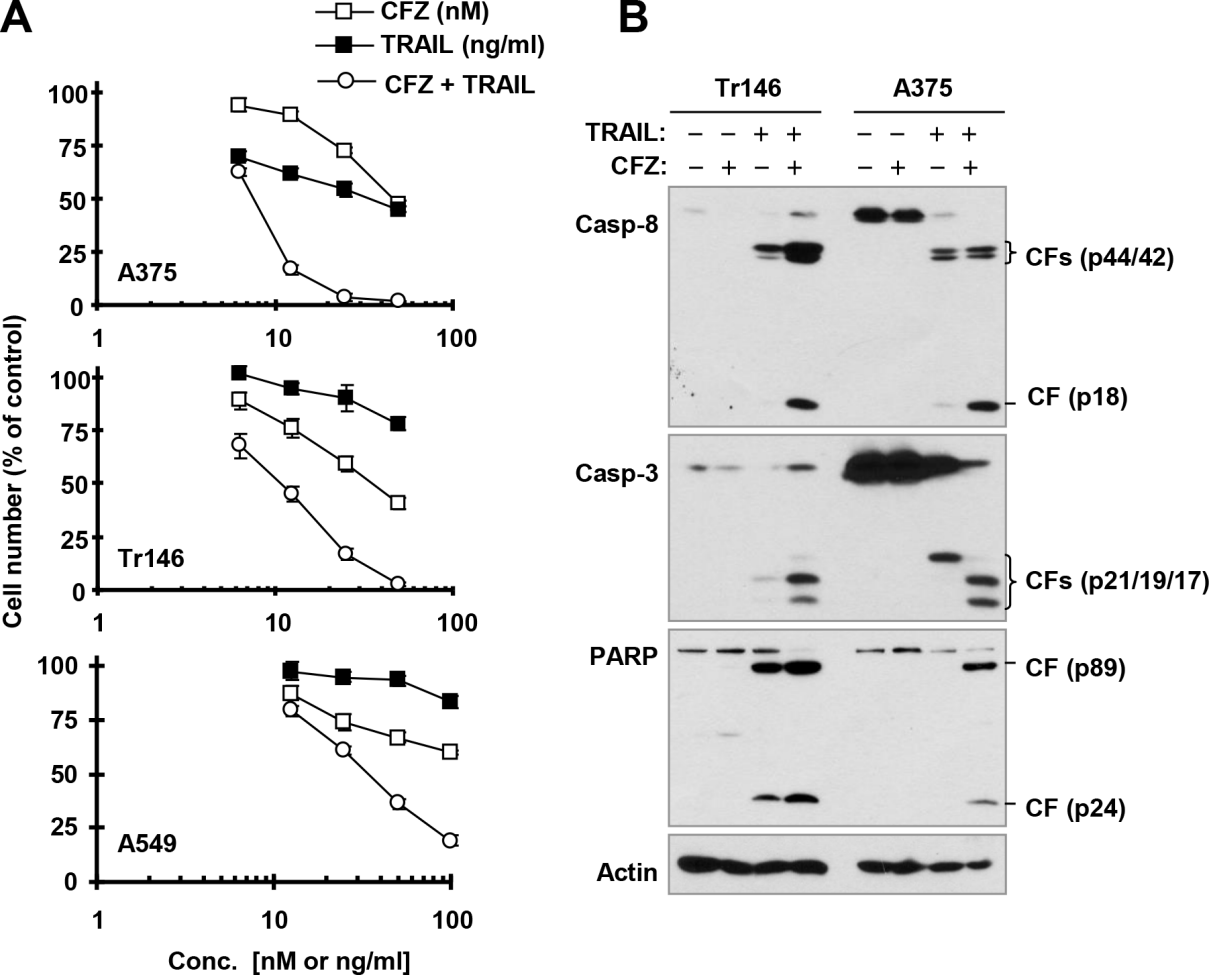
The combination of CFZ and TRAIL augments killing of cancer cells (A) with enhanced activation of caspases (B) A, The indicated cancer cell lines were treated with different concentrations of CFZ alone, TRAIL alone, and the combination of CFZ and TRAIL for 24 h and then subjected to estimation of cell number with the SRB assay. The data are means ± SDs of four replicate determinations. B, The indicated cell lines were treated with 50 nM CFZ alone, 25 ng/ml TRAIL alone or their combination for 24 h and then subjected to preparation of whole-cell protein lysates and subsequent Western blot analysis. CF, cleaved fragment.

Given that CFZ increases cell surface levels of both DR5 and DR4, which are well known death receptors for the death ligand TRAIL, we speculated that CFZ would sensitize cancer cells to TRAIL-induced apoptosis. To test this hypothesis, we examined the effects of CFZ in combination with TRAIL on cell survival and apoptosis in several cancer cell lines that are relatively less sensitive to TRAIL. TRAIL alone or CFZ alone at the tested concentration ranges weakly decreased the survival of the tested cancer cell lines; however, the combination of CFZ and TRAIL was much more active than either single agent in decreasing cell survival (Figure 4A). The combination indexes (CIs) for most combinations were far lower than 1, indicating strongly synergistic effects on cancer cell killing. Consistently, the combination of CFZ and TRAIL was more potent than each single agent in inducing cleavage of caspase-8, caspase-3 and PARP, evidenced by higher levels of cleaved forms in cells treated with the combination than in cells treated with either CFZ or TRAIL alone (Figure 4B). These data further support that the combination of CFZ and TRAIL enhances apoptosis in cancer cells.

**Figure 5:**
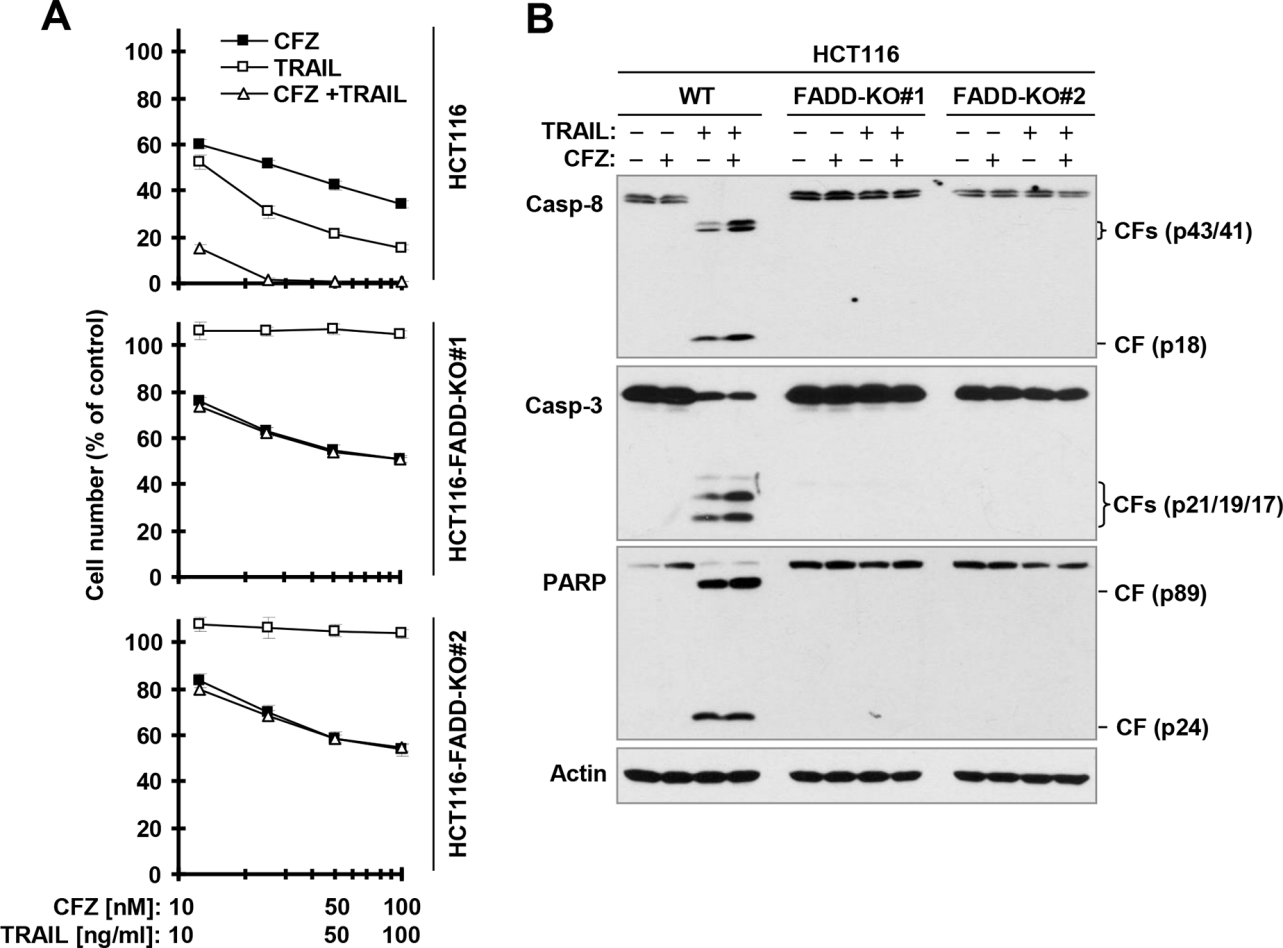
FADD deficiency abolishes augmented induction of apoptosis by the CFZ and TRAIL combination **A**, The indicated cancer cell lines were treated with different concentrations of CFZ alone, TRAIL alone, and the combination of CFZ and TRAIL for 24 h and then subjected to estimation of cell number with the SRB assay. The data are means ± SDs of four replicate determinations. **B**, The indicated cell lines were treated with 50 nM CFZ alone, 10 ng/ml TRAIL alone or their combination for 8 h and then subjected to preparation of whole-cell protein lysates and subsequent Western blot analysis. CF, cleaved fragment.

It is well known that TRAIL induces apoptosis exclusively through a FADD-dependent mechanism. To determine whether the enhanced cell-killing effect seen with the CFZ and TRAIL combination is simply due to enhancement of TRAIL-induced apoptosis, we compared the effects of the combination on cell death and caspase cleavage in WT and FADD-KO HCT116 cell lines. Indeed, the combination of CFZ and TRAIL enhanced cell death and cleavage of caspase-8, caspase-3 and PARP in WT HCT116, but not in FADD-KO cells (Figure 5). These data convincingly demonstrate that CFZ enhances TRAIL-induced apoptosis and cell-killing.

### DR5 upregulation contributes to CFZ-induced apoptosis

**Figure 6:**
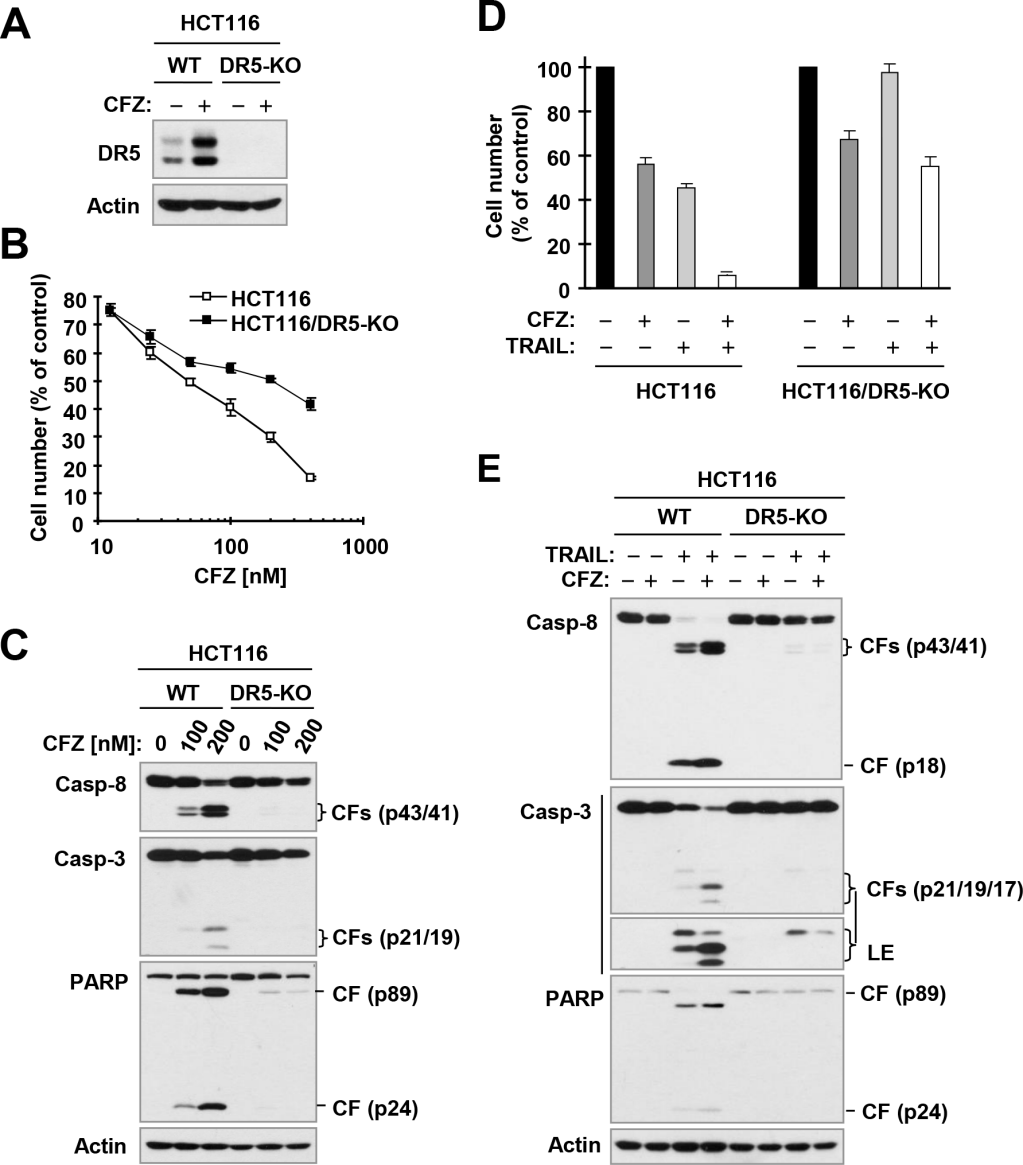
DR5 deficiency (A) protects cancer cells from undergoing apoptosis induced by CFZ (A and C) or CFZ and TRAIL combination (D and E) A, Detection of DR5 expression by Western blotting. B and D, The indicated cancer cell lines were treated with different concentrations of CFZ (B) or with 25 nM CFZ, 25 ng/ml TRAIL or the combination of CFZ and TRAIL (D). After 24 h, the cells were subjected to estimation of cell number with the SRB assay. The data are means ± SDs of four replicate determinations. C and E, The indicated cell lines were treated with different concentrations of CFZ (C) or with 50 nM CFZ alone, 15 ng/ml TRAIL alone or their combination (E) for 24 h (C) or 8 h (E) and then subjected to preparation of whole-cell protein lysates and subsequent Western blot analysis. CF, cleaved fragment. LE, longer exposure.

To determine whether DR5 upregulation is involved in CFZ-induced apoptosis in human cancer cells, we compared the effects of CFZ on cell death and caspase activation in WT and DR5-KO HCT116 cells (Figure 6A). We found that DR5-KO cells were significantly less sensitive than WT cells to CFZ in the cell survival assay (Figure 6B). Moreover, CFZ strongly induced cleavage of caspase-8, caspase-3 and PARP in WT HCT116 cells, but did so only minimally in DR5-KO cells (Figure 6C). Hence these results clearly show that DR5 upregulation is a critical event for CFZ to induce apoptosis.

### DR5 upregulation contributes to CFZ-mediated enhancement of TRAIL-induced apoptosis

We further determined whether CFZ enhances TRAIL-induced apoptosis via upregulation of DR5 using the same isogenic cell lines. The combination of CFZ and TRAIL enhanced cell death and cleavage of caspase-8, caspase-3 and PARP, as indicated by the elevated levels of cleaved forms of these proteins in WT cells, but not in DR5-KO cells (Figure 6D and 6E). This effect was consistent with the results observed with TRAIL alone, i.e., TRAIL induced cell death and cleavage of caspase-8, caspase-3 and PARP with less effect in DR5-KO cells than in WT cells (Figure 6D and 6E). Together, these results clearly indicate that CFZ upregulates DR5 expression, leading to enhancement of TRAIL-induced apoptosis.

### CFZ increases DR5 expression through enhancing protein stability and gene transcription

**Figure 7:**
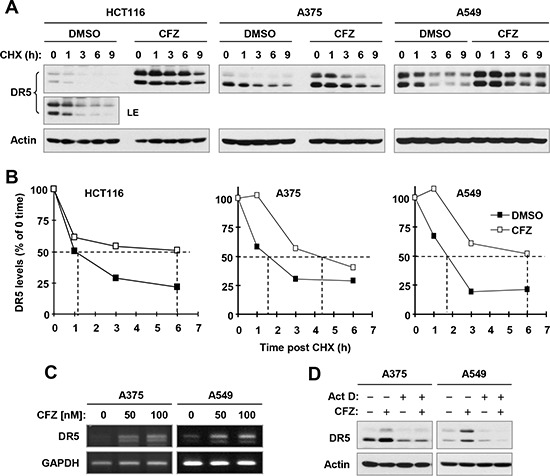
CFZ stabilizes DR5 protein (A and B) and increases DR5 transcription (C and D) in cancer cells A and B, The indicated cancer cell lines were exposed to 100 nM CFZ for 6 h. The cells were then washed with PBS 3 times and re-fed with fresh medium containing 10 μg/ml CHX. At the indicated times post-CHX, the cells were harvested for preparation of whole-cell protein lysates and subsequent Western blot analysis (A). Protein levels were quantified with NIH Image J Software and were normalized to actin (B). C, The indicated cell lines were treated with the given concentrations of CFZ for 8 h and then harvested for preparation of total cellular RNA and subsequent RT-PCR. D, The indicated cell lines were pre-treated with 2.5 μM actinomycin D (Act D) for 30 minutes and then co-treated with 100 nM CFZ for an additional 8 h. The cells were then harvested for preparation of whole-cell protein lysates and subsequent Western blot analysis. LE, longer exposure.

Since DR5 upregulation is strongly induced by CFZ across the tested cancer cell lines and plays a critical role in mediating CFZ-induced apoptosis and enhancement of TRAIL-induced apoptosis, we were interested in the mechanism by which CFZ upregulates DR5 expression. To this end, we examined the effect of CFZ on DR5 protein stability, since CFZ is a novel proteasome inhibitor. Hence, we conducted a cycloheximide (CHX) chase assay to compare degradation rates or half-lives of DR5 protein in the absence and presence of CFZ in 3 different cancer cell lines. The half-lives of DR5 in CFZ-treated cells were 4–6 h, compared with 1–2 h in DMSO-treated cells (Figure 7A and 7B), indicating that DR5 was degraded more slowly in CFZ-treated cells than in DMSO-treated cells. Hence, it is apparent that CFZ stabilizes DR5 protein.

In addition we found that CFZ also increased DR5 mRNA levels as evaluated with reverse transcription PCR (RT-PCR) in both A375 and A549 cells (Figure 7C). Moreover, the presence of the transcription inhibitor, actinomycin D, inhibited DR5 upregulation by CFZ (Figure 7D). Therefore, CFZ also increases DR5 transcription.

## DISCUSSION

In the present study, we have shown that CFZ effectively induces apoptosis and decreases the survival of different cancer cell lines. The induction of apoptosis by CFZ is at least in part due to activation of the extrinsic apoptotic pathway because deficiency of FADD, an essential component of the extrinsic apoptotic pathway, protected cells from CFZ-induced cell death and cleavage of caspases and PARP. Moreover, we have demonstrated that CFZ substantially enhances TRAIL-induced apoptosis, as evidenced by the synergistic induction of FADD-dependent apoptosis by the CFZ and TRAIL combination. To the best of our knowledge, this is the first study to indicate that CFZ induces and enhances extrinsic apoptosis in cancer cells.

Both DR5 and DR4 are death receptors through which TRAIL initiates extrinsic apoptosis. In this study, we found that total and cell surface levels of DR5 were strongly elevated by CFZ across the tested cancer cell lines, in comparison with a relatively modest increase in DR4. Hence, this study primarily focused on demonstrating the role of DR5 in mediating CFZ-induced apoptosis and enhancement of TRAIL-induced apoptosis and the mechanism accounting for CFZ-induced DR5 upregulation. Using genetically-manipulated DR5-deficient cells, we have robustly demonstrated for the first time that DR5 upregulation is a critical event that mediates CFZ-induced apoptosis and enhancement of TRAIL-induced apoptosis, since DR5 deficiency substantially protected cells from undergoing apoptosis induced by either CFZ or the CFZ and TRAIL combination. However, we cannot rule out that DR4 upregulation also contributes to CFZ-induced apoptosis and enhancement of TRAIL-induced apoptosis in a given cell line (e.g., A375).

Ubiquitination and proteasomal degradation is a key post-translational mechanism that regulates protein levels and biological functions including apoptosis [16]. It has been suggested that DR5 is regulated by this mechanism [17]. Since CFZ primarily functions as a proteasome inhibitor, it is reasonable to determine whether CFZ elevates DR5 levels by suppressing its degradation. Indeed, we demonstrated that CFZ treatment delayed the degradation of DR5 in a CHX chase assay (Figure 7). Therefore we conclude that stabilization of DR5 protein is an important mechanism by which CFZ upregulates DR5 expression. DR5 induction can also occur at transcriptional level [18–21]. In this study, we observed that CFZ also increased DR5 mRNA levels (Figure 7C) and inhibition of transcription with actinomycin D abolished the ability of CFZ to increased DR5 levels. Therefore, transcriptional regulation of DR5 expression is another mechanism contributing to the CFZ-induced upregulation of DR5. Clearly CFZ increases DR5 expression through enhancing both protein stabilization and gene transcription.

It is known that suppression of c-FLIP, a key negative regulator of the extrinsic apoptotic pathway, and/or Bcl-1 family members such as Mcl-1, Bcl-1 and Bcl-X_L_, which are negative regulators of the intrinsic apoptotic pathway, induces apoptosis or enhances TRAIL-induced apoptosis [22–24]. In this study, CFZ clearly increased the levels of both FLIP_S_ and Mcl-1 (Figure 3D). These findings are similar to previous observations that proteasome inhibition increased FLIP_S_ and Mcl-1 levels [25, 26]. Hence, elevation of these protein levels is likely to be a survival mechanism, which, however, can be overridden by upregulation of DR5 (and DR4) in cells exposed to CFZ, eventually resulting in apoptosis.

It is known that TRAIL rapidly induces apoptosis in a wide variety of transformed cells but is not cytotoxic in normal cells *in vitro* and *in vivo* [15, 27]. Thus, TRAIL is considered to be a tumor-selective, apoptosis-inducing cytokine and a promising new candidate for cancer therapy. In addition, agonistic anti-DR5 antibodies can induce DR5 trimerization, which triggers the extrinsic apoptotic pathway, and thus have great cancer therapeutic potential [28]. Unfortunately, certain cancer cells and tumors are resistant to apoptosis induced by TRAIL or DR5 agonistic antibody. Therefore, CFZ may be useful in combination with TRAIL or an agonistic anti-DR5 antibody to enhance the induction of apoptosis or overcome TRAIL resistance in human cancer cells.

## MATERIALS AND METHODS

### Reagents

CFZ was purchased from Selleck Chemicals (Houston, TX) and was dissolved in dimethyl sulfoxide (DMSO) at a concentration of 1 mM, and aliquots were stored at −80°C. Stock solutions were diluted to the desired final concentrations with growth medium just before use. CHX and actinoimycin D were purchased from Sigma Chemical Co. (St. Louis, MO). Human recombinant TRAIL was purchased from PeproTech, Inc. (Rocky Hill, NJ). Rabbit polyclonal anti-DR5 antibody was purchased from ProSci, Inc. (Poway, CA). Mouse monoclonal anti-DR4 antibody (B-N28) was purchased from Diaclone (Stamford, CT). Mouse monoclonal anti-caspase-3 was purchased from Imgenex (San Diego, CA). Rabbit anti-caspase-8 and anti-PARP antibodies were purchased from Cell Signaling Technology, Inc. (Beverly, MA). Mouse monoclonal anti-FLIP antibody (NF6) was purchased from Alexis Biochemicals (San Diego, CA). Rabbit polyclonal Mcl-1 and Bcl-X_L_ and mouse monoclonal Bcl-2 antibody were purchased from Santa Cruz Biotechnology, Inc. (Santa Cruz, CA). Rabbit polyclonal anti-β-actin antibody was purchased from Sigma Chemical Co.

### Cell lines and cell culture

A549 (lung cancer), H1299 (lung cancer), Tr146 (head and neck cancer) and SqCC/Y1 (head and neck cancer) cell lines were described previously [29, 30]. A375 (melanoma) cells were provided by Dr. J. Arbiser (Emory University, Atlanta, GA). HCT116 (colon cancer) and its isogenic FADD-KO and DR5-KO cell lines were provided by Dr. J. Yu (University of Pittsburgh, Pittsburgh, PA) and Dr. L. Zhang (University of Pittsburgh, Pittsburgh, PA), respectively. Except for A549 cells, which were authenticated by Genetica DNA Laboratories, Inc. (Cincinnati, OH) through analyzing short tandem repeat DNA profile, other cell lines were not authenticated. Cells were grown in monolayer culture in RPMI 1640 with glutamine, DMEM/F12 or McCoy's 5A modified medium supplemented with 5% fetal bovine serum at 37°C in a humidified atmosphere consisting of 5% CO_2_ and 95% air.

### Cell survival and apoptosis assays

Cells were seeded in 96-well cell culture plates and treated the next day with the tested agents. Viable cell numbers were determined using sulforhodamine B (SRB) assay as described previously [29]. CI was calculated using the CompuSyn software (ComboSyn, Inc.; Paramus, NJ) to indicate drug interaction (e.g., synergy). Apoptosis was evaluated primarily by detecting caspase and PARP cleavage with Western blot analysis as described below. We also used a Cell Death Detection ELISA^Plus^ kit (Roche Molecular Biochemicals, Indianapolis, IN) to detect DNA fragments according to the manufacturer's instructions as an additional indicator of apoptosis.

### Western blot analysis

Preparation of whole-cell protein lysates and the procedures for Western blotting were described previously [31].

### Detection of cell surface death receptors

The procedure for direct antibody staining and subsequent flow cytometric analysis of cell surface proteins was described previously [25]. Mean fluorescence intensity (MFI), which represents antigenic density on a per cell basis, was used to represent the cell surface death receptor expression level.

### Gene silencing with siRNA

The control and caspase-8 siRNA were the same as described previously [31]. siRNA transfection was performed as previously described [19, 32].

### Detection of DR5 mRNA expression

DR5 mRNA was detected with RT-PCR as described previously [33].
